# Aromatic C–H bond functionalization through organocatalyzed asymmetric intermolecular aza-Friedel–Crafts reaction: a recent update

**DOI:** 10.3762/bjoc.19.72

**Published:** 2023-06-28

**Authors:** Anup Biswas

**Affiliations:** 1 Department of Chemistry, Hooghly Women’s College, Vivekananda Road, Pipulpati, Hooghly - 712103, WB, India

**Keywords:** asymmetric, aza-Friedel–Crafts reaction, H-bonding, organocatalysis, stereoselectivity

## Abstract

The aza-Friedel–Crafts reaction allows an efficient coupling of electron-rich aromatic systems with imines for the facile incorporation of aminoalkyl groups into the aromatic ring. This reaction has a great scope of forming aza-stereocenters which can be tuned by different asymmetric catalysts. This review assembles recent advances in asymmetric aza-Friedel–Crafts reactions mediated by organocatalysts. The mechanistic interpretation with the origin of stereoselectivity is also explained.

## Introduction

The ease of a chemical transformation depends on the thermodynamic instability of a chemical bond owing to its fast cleavage under mild reaction conditions. A C–H bond is thermodynamically stable and possesses a high bond dissociation energy opposing the bond to easy chemical transformation. Therefore, harsh reaction conditions and the necessity of an external activator like catalysts are common prerequisites for processes involving C–H bond breaking. Among different types of C–H bonds, an aromatic C–H bond is even more inert rendering this type of bond functionalization more difficult. Herewith the term “bond functionalization” is defined as the cleavage of an existing bond with substitution by another bond. Aromatic C–H bond functionalizations have gained considerable attention by organic chemists because of the strategic importance of this process as well as the ability to synthesize functionalized aromatic molecules in a straightforward way. Many organic name reactions have been discovered utilizing the C–H bond functionalization concept [1].

Metals were exclusively explored to assist substitutions of aromatic C–H bonds by other bonds and this area of research is more than a century old. However, many disadvantages are associated with metal-mediated organic transformations including harsh reaction conditions (e.g., high temperature) and toxic solvents. With the tremendous progress in organic chemistry over the last few decades, metal catalysis has been increasingly and successfully replaced by organocatalysis, i.e., accelerating the rate of chemical transformations by using small organic molecules as catalysts. Although being discovered more than 100 years ago, the concept became increasingly accepted and popular only by the last decade of the last century [2–3].

Nowadays, organocatalysis is especially applied to asymmetric synthesis and a huge number of organocatalysts has been introduced in last three decades for the asymmetric synthesis of acyclic, carbocyclic, heterocyclic, and polycyclic molecular architectures with high molecular complexity. In particular, asymmetric organocatalysis plays a pivotal role in the construction of optically active, bioactive, and natural products. The main advantages of organocatalyzed stereoselective reactions include mild reaction conditions and the use of a sole catalyst without the need of other chiral ligands [4–5]. In these reactions, stereoinduction in the products is achieved by the chiral environment present in the catalyst itself. Depending upon the reactivities, organocatalysts can be categorized into two major divisions: 1) covalent bonding and 2) noncovalent bonding catalysts. A covalent bonding organocatalyst reacts with a substrate to form an activated chiral intermediate which undergoes a stereoselective reaction with another reagent. A noncovalent bonding catalyst usually assembles the reaction partners in a highly ordered three dimensional transition state through noncovalent interactions (like H-bonding, π–π interactions) thus promoting the stereoselective reaction. Examples of covalent bonding organocatalysts are amines [6–7], N-heterocyclic carbenes [8–9], phosphines [10], amidines [11], isothioureas [12–13], whereas thioureas [14–15], ureas [16], phosphoric acids [17–18], and squaramides [19–20] fall into the second category.

The Friedel–Crafts reaction, discovered by Charles Friedel and James Crafts in 1877 allows the aromatic C–H bond functionalization through the formation of a new C–C bond [21]. The reaction requires an electrophilic reagent/intermediate present in the reaction system on which an electrophilic attack by the π-electron cloud of the aromatic ring can occur spontaneously to form a dearomatized species. The latter is rearomatized in a succeeding step with the elimination of a H^+^ ion to form the functionalized aromatic moieties. The aza-Friedel–Crafts reaction is a subclass of the originally reported transformation that couples an imine with an aromatic system allowing for a facile incorporation of an alkylamine functionality into the aromatic system. Like the classical Friedel–Crafts reaction, the aza-Friedel–Crafts reaction also requires the presence of a Lewis acid catalyst for rate acceleration. The reaction can be very easily modulated by different Lewis acidic metallic compounds which effectively form a coordinate bond by accepting the lone pair of electrons of the imine nitrogen to a suitable vacant orbital of the metal center, thus enhancing the electrophilicity of the imine carbon atom by imparting a positive character on the adjacent heteroatom [22–23].

With the advent of different types of organocatalysts, the aza-Friedel–Crafts reaction has also been explored under the influence of organocatalysis. However, here organocatalysts act as Brønsted acids which form noncovalent interactions (H-bonding) with the imine nitrogen to enhance the electrophilicity of the imine component. In addition, by selecting suitable imine components, asymmetric products containing a nitrogen-substituted stereocenter can be obtained. Chiral organocatalysts can easily influence asymmetric aza-Friedel–Crafts reactions. The asymmetric induction is attributed to the formation of a chiral complex through a noncovalent interaction with the imine nitrogen and the catalyst which selectively blocks one face of the imine’s plane. This forces the nucleophile to approach from the opposite face thus imparting stereoselectivity into the products.

**Scheme 1:**
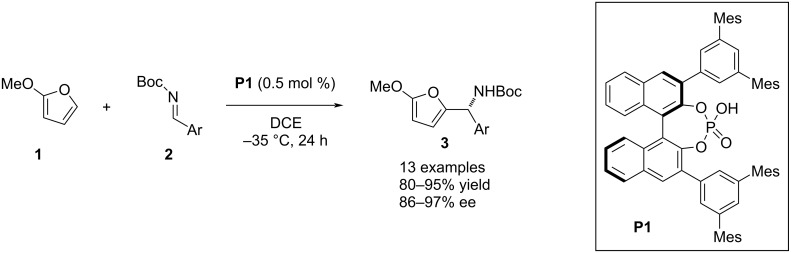
First organocatalyzed asymmetric aza-Friedel–Crafts reaction.

The first organocatalyzed asymmetric aza-Friedel–Crafts protocol was published by Terada and co-workers in 2004. In this methodology, a 1,1’-bi-2-naphthol (BINOL)-derived chiral phosphoric acid **P1** was used as the catalytic reagent to couple 2-methoxyfuran (**1**) and *N*-Boc-protected aldimines **2** to incorporate an aza-tertiary stereocenter into the 2’ position of the heteroaromatic products **3** (Scheme 1) [24].

This review summarizes the recent advances (2018 till date) on organocatalyzed asymmetric aza-Friedel–Crafts reactions. The examples have been segmented according to the different types of catalysts.

## Review

### Phosphoric acids

Chiral phosphoric acids have been envisaged as versatile organocatalysts for various asymmetric chemical transformations. These compounds play a dual role in the catalytic cycle due to their intrinsic Brønsted acidity and the ability to H-bond formation. Organophosphoric acids can perform as both H-bond acceptors and donors. 1,1’-Bi-2-naphthol (BINOL) and 1,1’-spirobiindane-7,7’-diol (SPINOL)-derived phosphoric acids with different substituents in the 2,2’-positions of the aromatic framework have been extensively explored as axially chiral catalysts in the field of asymmetric transformations including aza-Friedel–Crafts reactions.

**Scheme 2:**
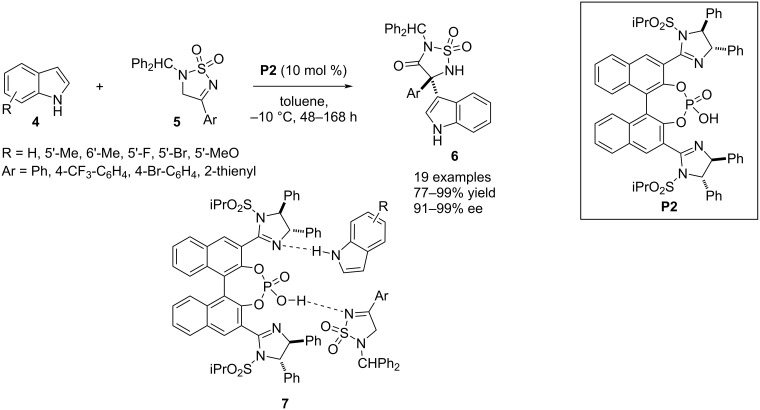
Aza-Friedel–Crafts reaction between indoles and cyclic ketimines.

In 2018, Nakamura and co-workers designed an aza-Friedel–Crafts process between indoles **4** and cyclic *N*-sulfonyl ketimines **5**. The authors employed the BINOL-based chiral phosphoric acid **P2** bearing two imidazoline moieties at the *ortho*-positions as the catalyst which activates both reactants through H-bonding where the NH group of the nucleophile performs as an H-bond donor towards the imidazoline nitrogen and the electrophile acts as H-bond acceptor from the OH group of the catalyst. These interactions rearrange the three molecules in a chiral pocket as shown by transition state **7**, favoring stereoinduction in the products through C3-functionalization of the indole (Scheme 2) [25].

**Scheme 3:**
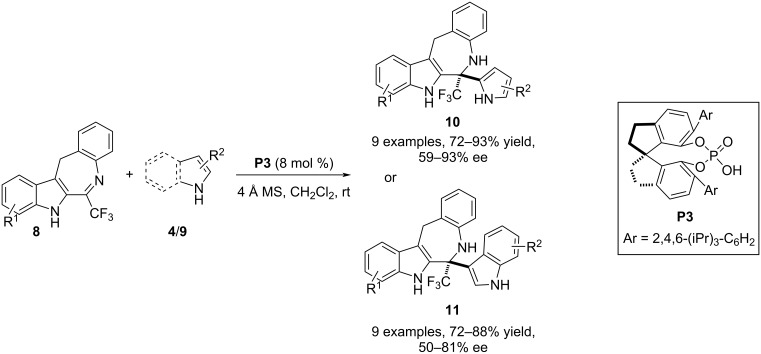
Aza-Friedel–Crafts reaction utilizing trifluoromethyldihydrobenzoazepinoindoles as electrophiles.

In 2018, Lin and co-workers deployed pyrroles **9** in an aza-Friedel–Crafts reaction with trifluoromethyldihydrobenzoazepinoindoles **8** to achieve the aromatic electrophilic substitution at the C2 position of the pyrrole ring. A further extension of the scope of this process was achieved through the C3–H functionalization of indole derivatives **4**. The nucleophile favors the attack at the imine carbon included in the seven-membered ring of compound **8** to generate an aza-quaternary stereocenter containing trifluoromethyl, pyrrole/indole, and benzoazepinoindole moieties. Stereoselectivity in the products **10**/**11** was achieved by using the chiral spirocyclic phosphoric acid catalyst **P3** which, through H-bonding interactions with the nucleophile and the electrophile, forces the nucleophile to approach the C=N plane from the *Re* face. In general, enantiocontrol with pyrroles was better than with indoles (Scheme 3) [26].

**Scheme 4:**
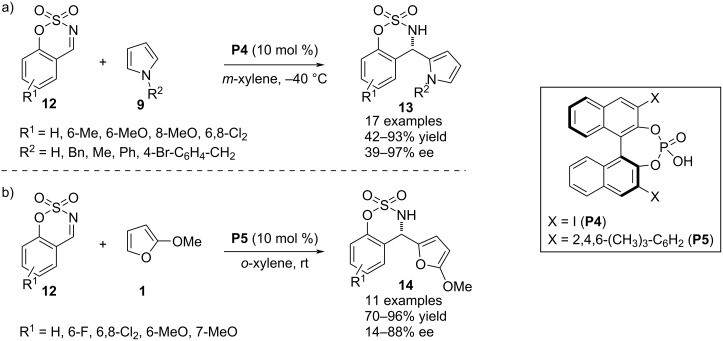
Aza-Friedel–Crafts reaction utilizing cyclic *N*-sulfimines as electrophiles.

In 2018, Kim and co-workers developed an aza-Friedel–Crafts protocol involving pyrroles **9** as the π-nucleophile in combination with cyclic *N*-sulfimines **12**. The chiral phosphoric acid **P4** was used to catalyze the introduction of a pyrrole-substituted aza-quaternary stereocenter in cyclic sulfamidate derivatives. *N*-Alkyl and *N*-benzyl-substituted pyrroles responded to the process with appreciable enantioefficiency. However, pyrrole was not proved to be the efficient substrate in terms of stereocontrol [27] (Scheme 4a). In the very next year, pyrrole was successfully replaced by 2-substituted furans **1** as the aromatic reacting partner with imines **12** to execute the asymmetric aza-Friedel–Crafts process modulated by the chiral phosphoric acid **P5** as the catalyst. A major concern of this process was the reduced aromatic character of the furan ring and the C2 methoxy-substituted substrate was exclusively employed to make the aromatic ring sufficiently electron rich. The substrate scope was mainly attributed to alterations of the substituents on the benzene ring of imines **12** (Scheme 4b) [28].

**Scheme 5:**
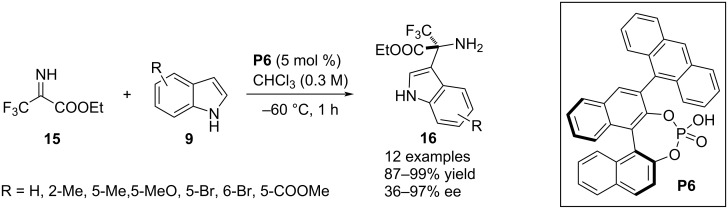
Aza-Friedel–Crafts reaction involving N-unprotected imino ester as electrophile.

In 2018, Morimoto, Ohshima and co-workers reported an aza-Friedel–Crafts process for the functionalization of the C3–H bond in indoles **9** in the presence of BINOL-derived chiral phosphoric acid **P6** as the catalytic agent. They utilized trifluoromethyl ester-substituted N-unprotected imine **15** as the potential electrophile to install an aza-quaternary stereocenter in the C3 position. The products **16** were achieved with excellent enantioselectivites which were attributed to an attractive interaction between the indole ring and the anthracene substituent of the catalyst’s framework (Scheme 5) [29].

**Scheme 6:**
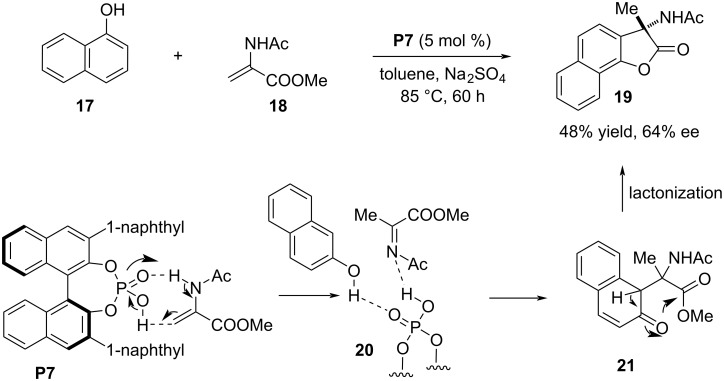
Aza-Friedel–Crafts and lactonization cascade.

In 2018, Piersanti and co-workers developed a phosphoric acid-catalyzed cascade reaction proceeding through aza-Friedel–Crafts reaction and lactonization steps. Main focus of this article was to demonstrate a racemic process between α-naphthol or phenol derivatives and in situ-generated *N*-acetyl ketimine from methyl 2-acetamidoacrylate (**18**) in the course of preparing 3-NHAc-naphthofuran or benzofuran analogues. The achiral phosphoric acid (PhO)_2_P(O)OH was the catalytic reagent to execute the process delivering the products with low to moderate chemical yields. Attempts to make the process stereoselective, a series of chiral phosphoric acid catalysts were screened in the model reaction between α-naphthol (**17**) and methyl 2-acetamidoacrylate (**18**) but promising selectivity was not achieved. The highest enantiomeric excess of 64% was obtained in the presence of **P7** as the catalyst (Scheme 6) [30].

**Scheme 7:**
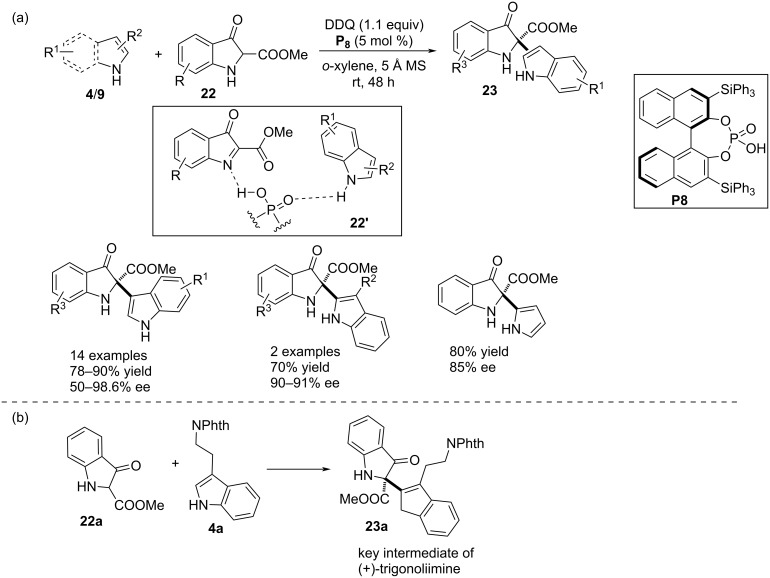
One-pot oxidation and aza-Friedel–Crafts reaction.

In 2018, Reddy and co-workers developed a one pot protocol comprising oxidation and an enantioselective aza-Friedel–Crafts addition. In the first step, the DDQ-promoted oxidation of 3-indolinonecarboxylate **22** generated indolenines that performed as the potential electrophiles towards indoles **4**. The chiral catalyst effectively assembled the reacting partners in a chiral transition state through H-bonding interactions to facilitate a highly face-selective nucleophilic attack by π-nucleophile to the cyclic imine (see transition state **22’** in Scheme 7a). The BINOL-derived chiral phosphoric acid **P8** was employed as the asymmetric organocatalyst for this transformation to construct the heterodimerized products **23** framed with an aza-quaternary stereocenter. Indole derivatives without any substitution in the heterocyclic ring participated in the reaction through the C3 position smoothly providing the products with appreciable yields and enantiocontrol. Two examples were demonstrated with 3-alkyl-substituted indoles which effectively attacked the electrophile through the C2 position. The reaction was even compatible with pyrroles (Scheme 7a). The utility of this methodology was successfully demonstrated by the synthesis of product **23a**, the key intermediate of natural product (+)-trigonoliimine (Scheme 7b) [31].

**Scheme 8:**
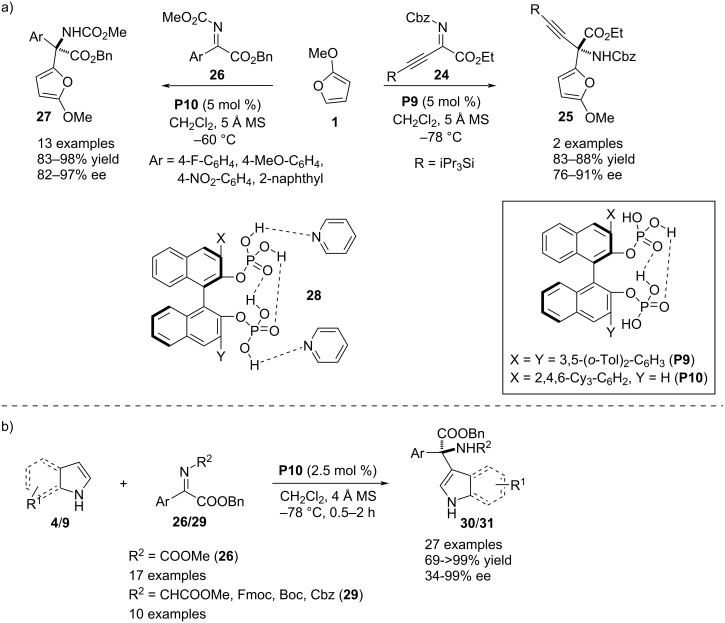
*C*_1_ and *C*_2_-symmetric phosphoric acids as catalysts.

In 2018, Ishihara and co-workers developed a novel *C*_2_ and *C*_1_-symmetric bisphosphoric acid-catalyzed asymmetric aza-Friedel–Crafts reaction. Both catalysts showed intramolecular H-bonding causing a sharp increase in Brønsted acidity of free OH groups and prevention of catalyst dimerization. The *C*_2_-symmetric **P9** promoted the reaction between 2-methoxyfuran (**1**) and β,γ-alkynyl-α-imino esters **24** to effect a C–C bond formation at the C2’ position of the heterocyclic ring. Only two examples were shown by varying the alkynyl substituent. The authors further extended the scope by studying the reaction between 2-methoxyfuran (**1**) and aryl-α-ketimino ester **26** to activate the C2’–H bond in **1**. The *C*_1_-symmetric catalyst **P10** was the optimal catalyst for the second reaction furnishing the products with excellent chemical yields and enantioselectivities. To understand the activities of the catalysts, the authors were able to obtain X-ray crystallographic data of the pyridine–catalyst complex which showed two intramolecular H-bonding interactions in the molecular framework of the catalyst where two free OH groups were engaged in interactions with the pyridine. This data clearly indicates the activation of the reaction components through H-bonding engagement with free hydroxy groups of the catalysts also favoring stereoselective addition (see structure **28** in Scheme 8a) [32]. Two years later, the same research group utilized the *C*_1_-symmetric catalyst **P10** for the functionalization of the C3–H bond of indole **9** through aza-Friedel–Crafts reaction with aryl-α-ketimino esters **26**/**29**. They also utilized unsubstituted and 2,3-disubstituted pyrroles **9** as π-nucleophile towards the same electrophiles to incorporate an amine-substituted quaternary stereocenter at the C2’ position (Scheme 8b) [33].

**Scheme 9:**
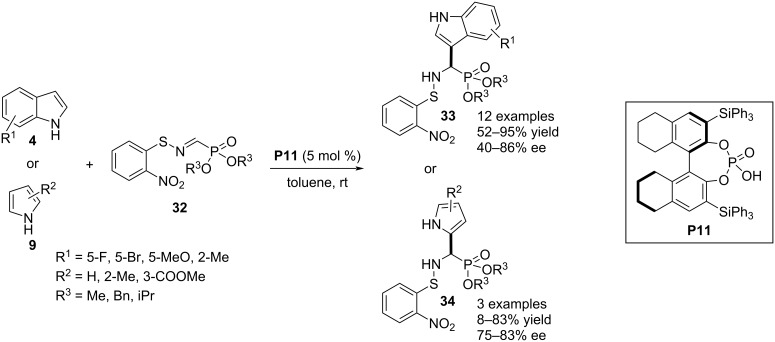
Aza-Friedel–Crafts reaction using Nps-iminophosphonates as electrophiles.

In 2019, Inokuma, Yamada and co-workers reported the C3–H bond functionalization of indoles **4** through aza-Friedel–Crafts reaction utilizing *N*-*o*-nitrophenylsulfenyl (Nps)-iminophosphonates **32** as electrophiles. The chiral phosphoric acid **P11** was used as H-bonding catalyst to impart stereoselectivities into the products, i.e., α-3-indolyl-α-aminophosphonic acids **33**. The reaction was also well compatible with pyrroles **4** proceeding through C2–H substitution. With C2-substituted pyrrole, the electrophile enters into the C5 position (Scheme 9) [34].

**Scheme 10:**
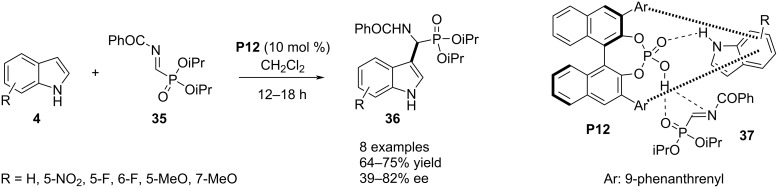
Aza-Friedel–Crafts reaction between indole and α-iminophosphonate.

In 2019, Palacios, Vicario and co-workers documented an aza-Friedel–Crafts reaction between indole **4** and α-iminophosphonate **35**. The reaction functionalized the C3 position of the heterocyclic ring with an α-aminophosphonate group. Chiral phosphoric acid **P12** was the stereoselectivity inducer in the products **36** as explained by π–π stacking and H-bonding interactions between the catalyst and the substrates (see transition state **37** in Scheme 10). The presented substrate scope was not broad and poor to moderate enantioselectivities were obtained. Indoles with a substituent in the carbocyclic ring required shorter reaction times to accomplish in comparison to C2-substituted indoles. The authors also tried the reaction with C3-substituted indoles to functionalize the C2 position. However, a very low enantioselectivity was achieved in the latter case (Scheme 10) [35].

**Scheme 11:**
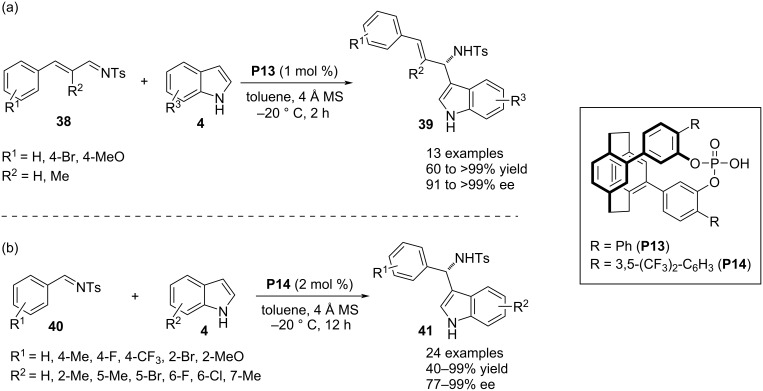
[2.2]-Paracyclophane-derived chiral phosphoric acids as catalyst.

Lin and co-workers designed a planar chiral phosphoric acid containing a [2.2]paracyclophane moiety that efficiently catalyzed the aza-Friedel–Crafts reaction between indole **4** and *N*-tosyl vinylaldimines **38** to functionalize the C3–H bond of the heterocyclic ring. The authors tried six such catalysts by varying the aromatic substituents, among which **P13** was proved to be the best one in terms of both yields and enantioselectivity. The catalyst **P13** was an even far superior catalyst than conventional BINOL and SPINOL-derived phosphoric acids. The substrate scope was investigated by varying substituents in the carbocyclic ring of indole **4**. Changing the β-aryl and α-substituents in the styryl-derived aldimines further expanded the substrate scope. Only 1 mol % catalyst loading was sufficiently efficient to deliver the enantioenriched products (Scheme 11a). The compatibility of the reaction was further explored by using *N*-tosyl arylaldimines **40** as the electrophilic partner to afford (aryl)(indolyl)methanamines **41** with high enantioselectivities. In this case, **P14** was identified as the optimal catalyst (Scheme 11b) [36].

**Scheme 12:**
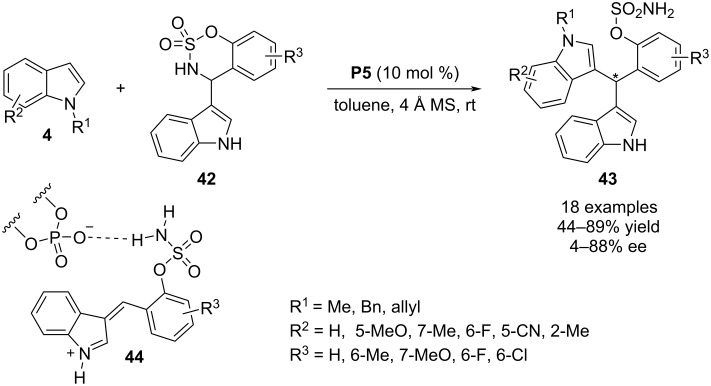
Aza-Friedel–Crafts reaction through ring opening of sulfamidates.

In 2019, Kim and co-workers reported a phosphoric acid-catalyzed enantioselective aza-Friedel–Crafts reaction between N-substituted indoles **4** and indol-3-ylsulfamidates **42**. The dual reactivity of catalyst **P5** initiated with the protonation of amidates **42** to generate intermediate **44** through ring cleavage. Then, the intermediate **44** was paired with the anionic conjugate base of catalyst **P5** and acts as electrophile to facilitate the conjugate Friedel–Crafts reaction involving C3 of indole **4** as the nucleophile. This reaction afforded (bis(indolyl)methyl)benzenesulfonamide derivatives **43** but no promising enantioselectivity was achieved for most of the products (Scheme 12) [37].

**Scheme 13:**
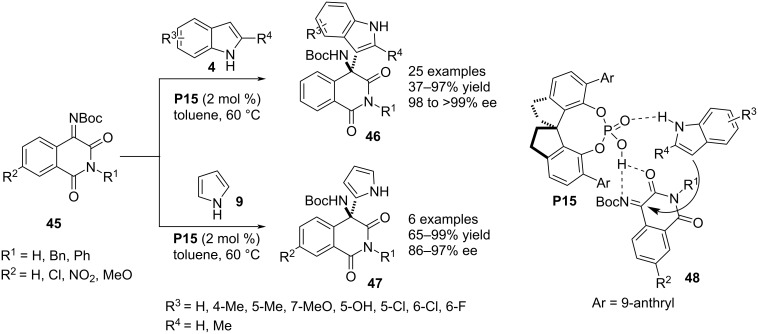
Isoquinoline-1,3(2*H*,4*H*)-dione scaffolds as electrophiles.

In 2019, You, Yuan and co-workers reported another enantioefficient aza-Friedel–Crafts reaction between N-unsubstituted pyrroles/indoles **4**/**9** and isoquinoline-1,3,4(2*H*)-trione-1-imines **45** installing an aza-quaternary stereocenter in isoquinoline-1,3(2*H*,4*H*)-dione frameworks **46**/**47**. The spinol-derived catalyst **P15** was applied for the asymmetric induction through H-bonding interaction with the NH group of the heteroarene and amide oxygen of **45** forcing the heteroarene to approach from the *Si*-face of the imine moiety predominantly (see transition state **48**) achieving high enantiocontrol for both heterocycles (Scheme 13) [38].

**Scheme 14:**
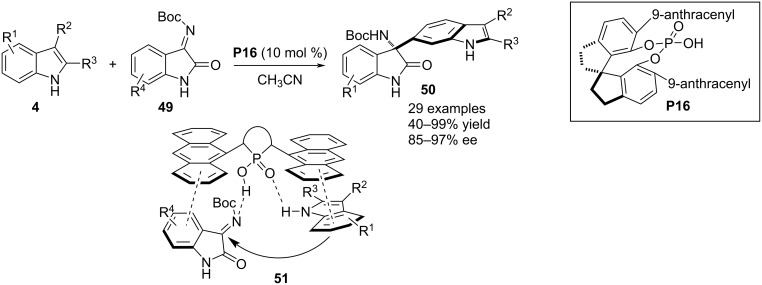
Functionalization of the carbocyclic ring of substituted indoles.

The carbocyclic ring in indoles is less reactive than the heterocyclic ring and hence the presence of an electron-donating functional group is crucial in the ring to activate it for aromatic electrophilic substitution processes. In 2019, Zhang and co-workers succeeded in the C6-selective aminoalkylation of 2,3-disubstituted indoles **4** without the presence of a directing group in the benzene ring. As the electron-demanding reaction partner, isatin-derived *N*-Boc-substituted ketimines **49** were employed which effectively functionalized the C6–H bond of substrate **4** to construct 3-oxindole derivatives **50** bearing an indole-substituted aza-quaternary stereocenter at its C3 position. 2,3-Dialkyl-substituted indoles having methyl or cycloalkyl substitutents of different ring sizes exclusively reacted as nucleophiles. Chiral phosphoric acid **P16** mediated the asymmetric transformation to regulate the stereochemical output of the quaternary stereocenter with good to excellent enantioselectivities. A resonance-assisted accumulation of negative charge on C6 enabled the carbon to add to the electrophile selectively from the *Re* face of the imine plane because of substrate–catalyst H-bonding interactions (see transition state **51**). Beside multiple noncovalent interactions, π–π stacking between the anthracenyl group of the catalyst framework and aromatic rings of both substrates was also responsible for the stereoselective addition (Scheme 14) [39].

**Scheme 15:**
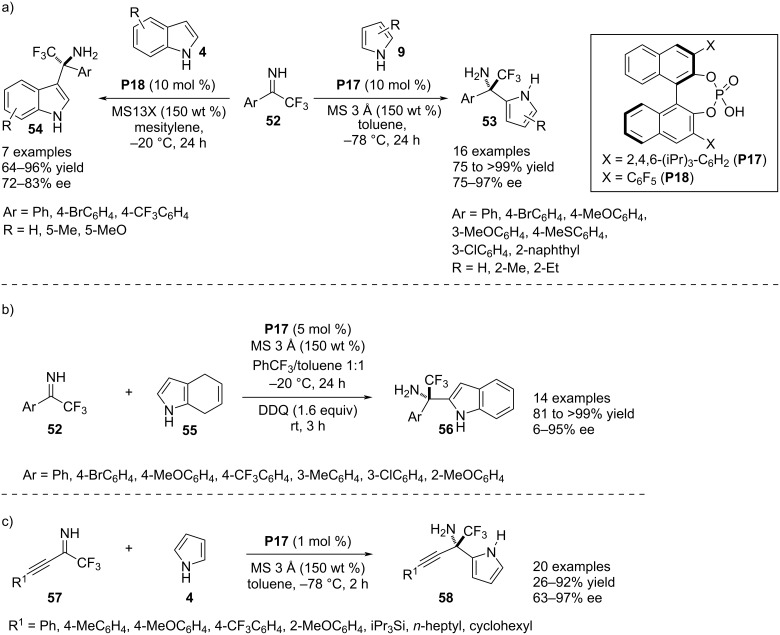
Aza-Friedel–Crafts reaction between unprotected imines and aza-heterocycles.

In 2019, Akiyama and co-workers developed a simple enantioselective aza-Friedel–Crafts process using unprotected pyrroles **9** and indoles **4** mediated by BINOL-derived chiral phosphoric acid catalysts **P17** and **P18**. The electrophile was the α-trifluoromethyl-containing imine **52** which directed the C2 functionalization in the pyrrole moiety with catalyst **P17** and a C3 substitution in indole derivatives using catalyst **P18** forming the trifluoromethylated aza-quaternary stereocenter. Excellent chemical yields and good to excellent levels of enantioselectivities in the products **53**/**54** were obtained by the chiral catalysts. The process was robust towards α-aryl- and α-trifluomethylimines and the substrate scope was mainly investigated by the variation of electron-donating groups in the aryl ring of the imines whereas amenability of this methodology was narrow for ring-substituted pyrroles and indoles (Scheme 15a) [40]. In the next year, the same research group reported another aza-Friedel–Crafts reaction between 4,7-dihydroindole (**55**) and N-unsubstituted trifluoromethylated ketimines **52** proceeding through C2 functionlization and follow up oxidation to provide 2-substitued indoles **56** which are typically difficult to obtain directly from unsubstituted indoles through electrophilic substitution. The process was catalyzed by the chiral phosphoric acid **P17** to install a quaternary stereocenter bearing primary amine and trifluoromethyl functionalities associated with appreciable enantiocontrol. The substrate scope was investigated by the variation of sterically and electronically divergent aryl substituents in the ketimines but the enantioselectivity was markedly lowered with sterically congested reactants (Scheme 15b) [41]. Very recently, Akiyama and co-workers demonstrated a C2-selective aza-Friedel–Crafts reaction of unmodified pyrroles **9** with (alkynyl)(trifluoromethyl)imines **57** catalyzed by the chiral phosphoric acid **P17**. This reaction produced an aza-quaternary stereocenter bearing 2-pyrrolyl, trifluoromethyl and alkynyl as other three substituents (Scheme 15c) [42].

**Scheme 16:**
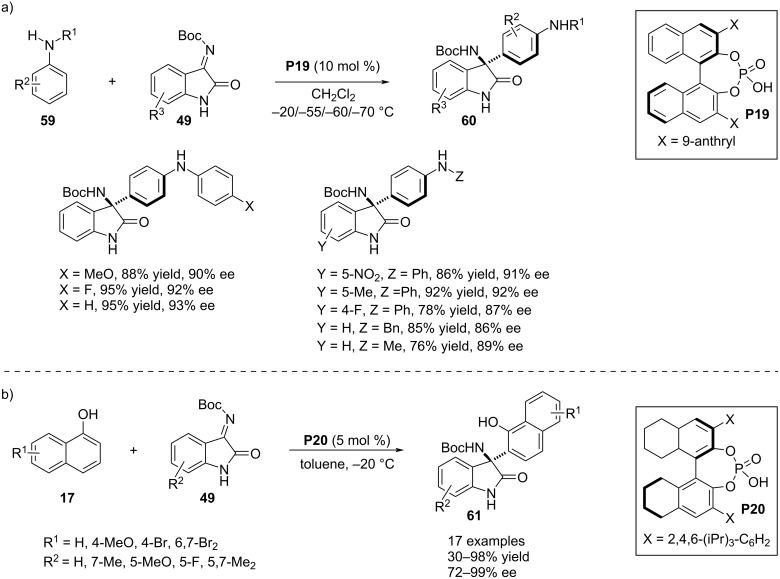
Anilines and α-naphthols as potential nucleophiles.

In 2020, a completely *para*-selective aza-Friedel–Crafts protocol with *N*-monosubstituted aniline derivatives **59** catalyzed by the chiral phosphoric acid **P19** was disclosed by Zhu, Zhang and co-workers [43]. The electrophilic aromatic substitution involved isatin-derived ketimines **49** as the electron-demanding partner to achieve this aromatic *p*-C–H bond functionalization framing an all substituted stereocenter at the C3 position of the oxindole scaffold in the products **60**. A very low reaction temperature (−55/−60 °C) was ideal to obtain the products with satisfactory enantioselectivities. The reaction was compatible with a broad range of substrates using *para*-substituted phenyl rings as the nitrogen substituents in anilinies **59**. Two examples were shown with *N*-benzyl and *N*-methyl-substituted anilines which afforded the desired products as well but an elevated temperature was required for these reactions. Further expansion of the substrate scope was achieved by altering functionalities with contrasting electronic and steric nature in the benzene ring of substrate **49**. Generally high enantioselectivities were obtained with *N*-aryl-substituted anilines **59** which decreased in case of *N*-alkyl-substituted anilines. This observation led to the development of a plausible transition state of the stereoselective electrophilic addition which included dual H-bonding interactions between both the substrates and the catalyst along with π–π interactions between the catalyst’s aryl group and the aryl substituent at the nitrogen in the aniline **59** (Scheme 16a) [43]. Recently, Fan and co-workers reported a chiral phosphoric acid **P20**-assisted enantioselective aza-Friedel–Crafts reaction between α-naphthols **17** and isatin-derived ketimines **49** to construct an aza-quaternary stereocenter at the C3 position of oxindole scaffolds **61** bearing a β-naphtholyl substituent (Scheme 16b) [44].

**Scheme 17:**
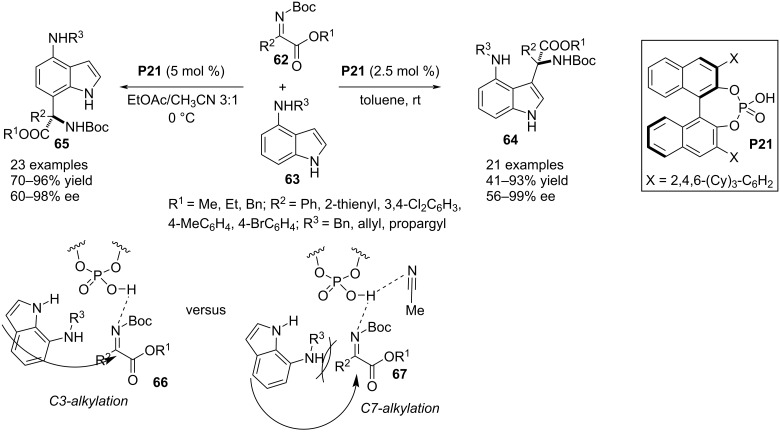
Solvent-controlled regioselective aza-Friedel–Crafts reaction.

In 2020, Meng, Chan, Zhao and co-workers reported another C3-selective aza-Friedel–Crafts reaction of 4-aminoindole derivatives **63** utilizing *N*-Boc-α-ketimino esters **62** as potential electrophiles. The chiral phosphoric acid **P21** catalyzed this process facilitating the formation of a quaternary stereocenter containing α-amino esters. Switching the solvent from non-polar to polar showed a regioselectivity shift to a C7 alkylation of the indole ring. The solvent-controlled regioselectivity switch of this aza-Friedel–Crafts reaction can be explained by the involvement of the polar solvent (acetonitrile) in the H-bonding with the catalyst thus creating a more hindered environment for a C3 alkylation, rather favoring the reaction through the less congested site (see transition states **66** and **67**, Scheme 17) [45].

**Scheme 18:**
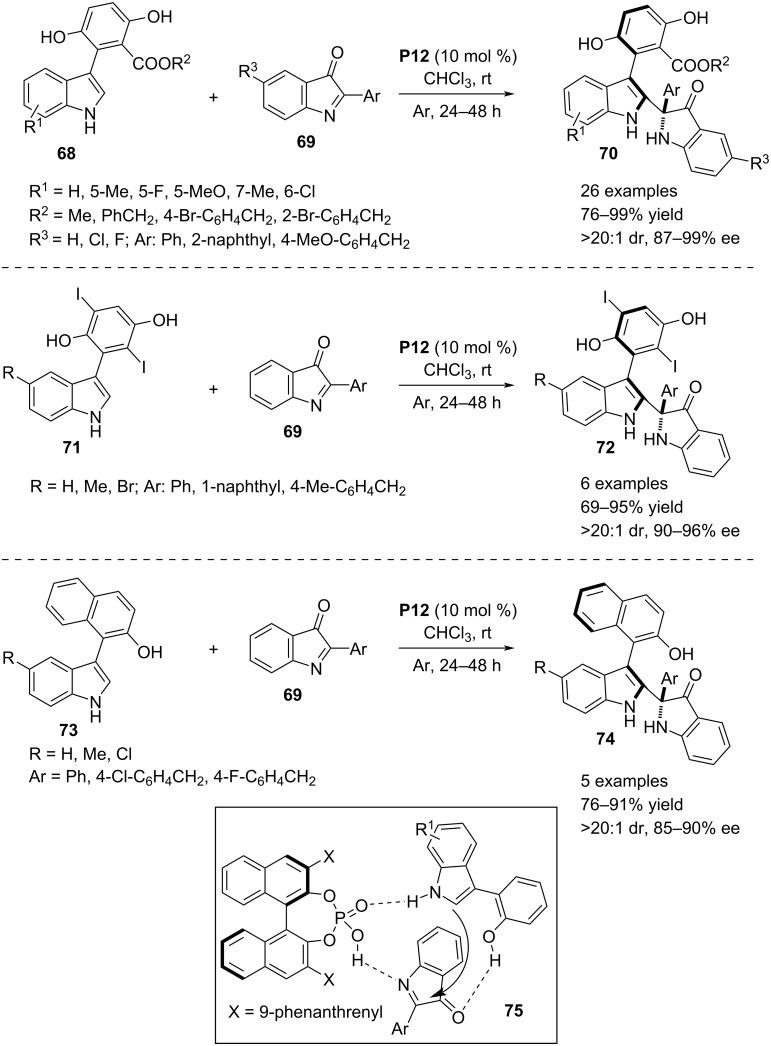
Generating central and axial chirality via aza-Friedel–Crafts reaction.

In 2020, Fu and co-workers developed a novel aza-Friedel–Crafts reaction between 3-arylindoles **68** and 2-aryl-3*H*-indol-3-ones **69** activating the C2–H bond of the heteroaromatic ring. Chiral phosphoric acid **P12** catalyzed this transformation generating a complex molecular topology of 2,3-disubstituted indoles bearing both axial and central chirality. The aza-Friedel–Crafts reaction would allow the nucleophile to selectively attack the C=N plane of the electrophile as directed by a triple hydrogen-bonded complex between the catalyst and the substrates (see transition state **75**, Scheme 18). This C–C-bond formation affords a 3-indolinone moiety bearing an aza-quaternary stereocenter at the C2 position. In addition, the reaction allows to obtain axially chiral products **70**/**72**/**74** through restriction of the C–C bond rotation around the heteroaryl and aryl moieties. For this purpose, sterically bulky substituents need to be present in the aryl ring attached to the C3 position of the starting indoles. The axial chirality was attributed to ester and phenolic OH groups at the *ortho*-positions of the aryl ring and an additional phenolic OH functionality at the *meta*-position (substrate **68**). Some more substrates were prepared by introducing a 2,5-diiodo-3,6-dihydroxyphenyl substitution at the C3 position of the indole ring (substrate **71**). The products were formed with high chemical yields and excellent diastereo- and enantioselectivities. A further expansion of the substrate scope was demonstrated by incorporating a β-naphthol ring as the C3 substituent of the indole moiety (substrate **73**). In all classes of bi(heteroaryl) substrates, a phenolic OH group at the *ortho*-position was crucial as it was involved in an intermolecular hydrogen bonding with the carbonyl oxygen of **69** in the ternary complex, thus bringing more rigidity in the three dimensional transition state (Scheme 18) [46].

**Scheme 19:**
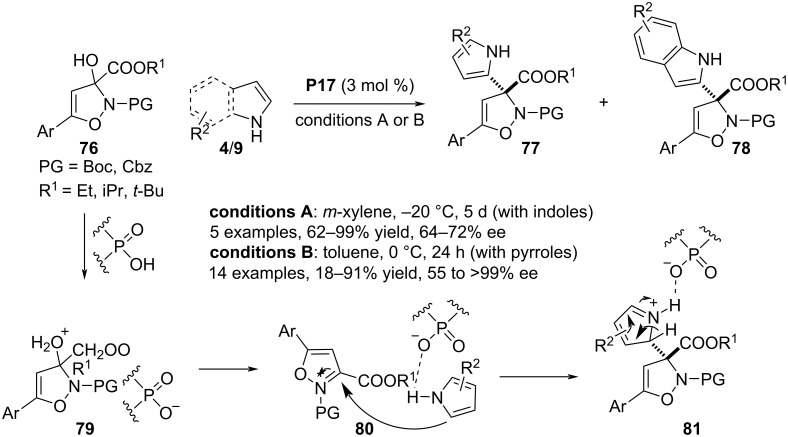
Reaction between indoles and racemic 2,3-dihydroisoxazol-3-ol derivatives.

In 2021, Chen and co-workers documented a chiral phosphoric acid **P17**-catalyzed aza-Friedel–Crafts process between racemic 2,3-dihydroisoxazol-3-ol derivatives **76** and pyrroles/indoles **4**/**9** allowing access to 2,3-dihydroisoxazoles **77**/**78** bearing an all-substituted stereocenter at the C3 position. A dual catalytic activity of the Brønsted acid catalyst was illustrated by the authors which was initiated with a smooth protonation of the OH group in **76** with a subsequnte dehydration to generate isoxazolium cation **80** paired with a phosphate anion. This chiral phosphate is engaged in H-bonding with the free NH of the heteroarene ring to ease the stereoselective 1,2-addition to in situ generate the cationic heterocyclic scaffold **81**. The reaction proceeded faster with pyrroles than with indole (Scheme 19) [47].

**Scheme 20:**
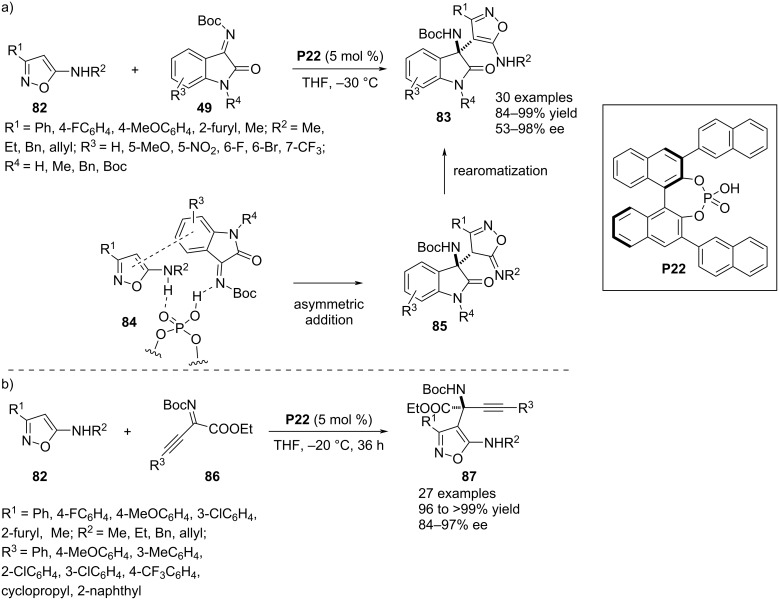
Exploiting 5-aminoisoxazoles as nucleophiles.

In 2021, Zhang and co-workers used 5-aminoisoxazole scaffolds **82** in an enantioefficient aza-Friedel–Crafts reaction with isatin-derived *N*-Boc ketimines **49**. A 2-oxindole-substituted aza-quaternary stereocenter was installed at the C4 position of the heteroaromatic ring in **83** and the enantioregulation was achieved by BINOL-derived chiral phosphoric acid **P22**. An amine functionality was crucial in the isoxazole ring to enhance the nucleophilicity of the adjacent carbon atom. In addition, the amine hydrogen forms an H-bond with the catalyst along with another hydrogen bond formed between the imine nitrogen of **49** and the catalyst’s OH group (see transition state **84**). These dual H-bonding interactions were assisted by a π–π interaction between the arene rings of both the electrophile and nucleophile that helped in the formation of a stereodefined transition state. The substrate scope was achieved by varying the substituents in the C3 position of the isoxazoles **82** and the carbocyclic ring substituents in ketimines **49**. Few more products were added to the library by altering the substituents of the amine in **82** and the ring nitrogen in **49** (Scheme 20a) [48]. The nucleophilcity of C3-substituted 5-aminoisooxazoles **82** was further utilized in another aza-Friedel–Crafts reaction with β,γ-alkynyl-α-ketimino esters **86** to provide *N*-Boc α-amino esters containing a quaternary stereocenter at the α-carbon. The chiral phosphoric acid **P22** was used as catalyst to introduce the aza-ester quaternary sterocenter in the molecular entities **87** with appreciable chemical yields and excellent enantioselectivities. One example was presented with a 5-aminoisothiazole motif that gave the product with much decreased yield (70%) and enantioselectivity (36% ee) (Scheme 20b) [49].

**Scheme 21:**
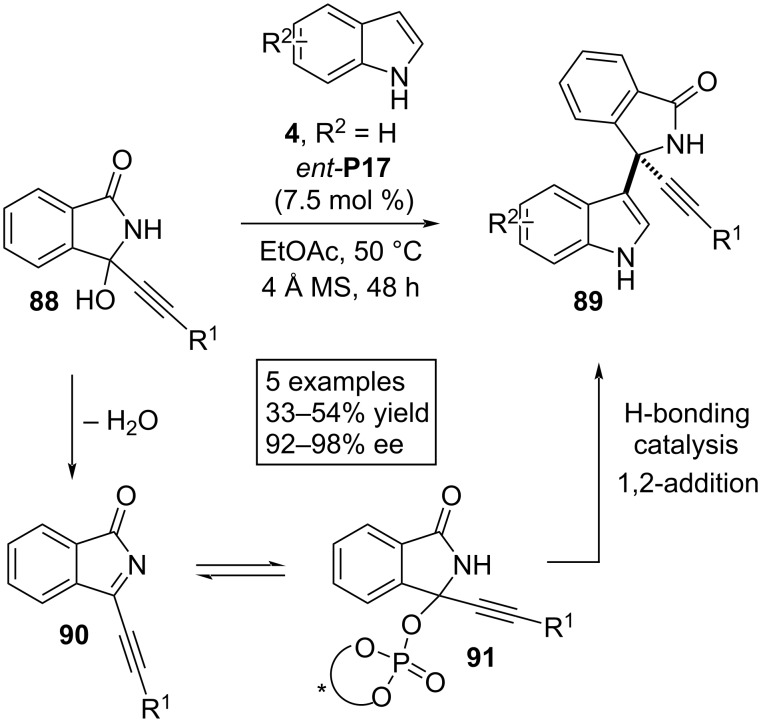
Reaction between unsubstituted indoles and 3-alkynylated 3-hydroxy-1-oxoisoindolines.

In 2022, Sun, Li and co-workers developed an aza-Friedel–Crafts technique involving 3-alkynylated 3-hydroxy-1-oxoisoindolines **88** as electrophiles in combination with unsubstituted indoles **4** in the presence of chiral phosphoric acid *ent*-**P17** as the catalytic agent. Facile dehydration of **88** was facilitated by the Brønsted acid to generate (*N*-acyl)(propargyl)imine **90** as intermediate which added to the deprotonated phosphoric acid to form phosphate ester **91** as the next intermediate through an equilibrium process. Then, 1,2-addition by the C3 position of the heteroarene ring to the acylimine intermediate afforded the 3-indolyl-substituted aza-quaternary stereocenter. Here the stereoselectivity was attributed to an H-bonding interaction between the catalyst and the substrates (Scheme 21) [50].

**Scheme 22:**
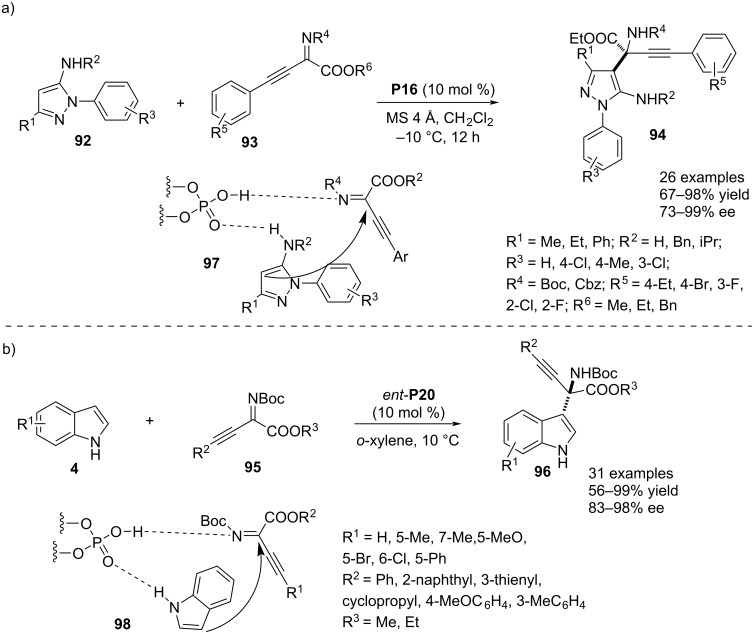
Synthesis of unnatural amino acids bearing an aza-quaternary stereocenter.

In 2022, Lin and co-workers reported an unusual aza-Friedel–Crafts reaction using *N*-aryl-5-aminopyrazoles **92** as potential π-nucleophiles in combination with β,γ-alkynyl-α-imino esters **93** acting as the electrophilic reagent. Chiral phosphoric acid **P16** was the catalytic agent to access a series of enantioenriched α-amino esters **94** containing 5-aminopyrazolyl and alkynyl substituents at the α-carbon. A library of products was prepared by varying different parts of both nucleophile and electrophile. The enantioselectivity of the reaction was an obvious result of a dual H-bonding interaction between the catalyst and both substrates where the imine nitrogen of **93** acted as H-bond acceptor and the amine functionality in **92** as H-bond donor to the catalyst (see transition state **97**, Scheme 22a) [51]. Recently, the same research group documented another aza-Friedel–Crafts reaction between indoles **4** and **95** that frames aza-quaternary stereocenter at the α-carbon of unnatural amino acid derivatives **96**. Enantiocontrol was rationalized by dual H-bonding interactions between both the reagents and the catalyst. The indole’s NH performed as the H-bond donor whereas the imine nitrogen of **95** was the H-bond acceptor towards the catalyst enabling a face-selective attack by the π-nucleophile to the electrophile C=N plane (see transition state **98**). The substrate scope comprised mainly varying aryl or heteroaryl-substituents at the alkyne moiety that imparted high degrees of enantioselectivities to the products (Scheme 22b) [52].

**Scheme 23:**
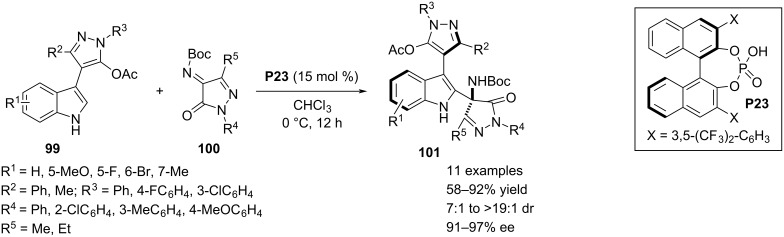
Atroposelective aza-Friedel–Crafts reaction.

In 2022, Huang and co-workers demonstrated an atroposelective construction of 3,4’-indole-pyrazole frameworks achieved through an asymmetric aza-Friedel–Crafts reaction. As substrate the authors chose the racemate of indole moiety **99** bearing a 5-acetyloxypyrazol substitution at the C3 position which was coupled with the pyrazolone-derived imine **100** to functionalize the C2–H bond of the indole ring. This aromatic electrophilic substitution also gave a quaternary aza-stereocenter in the pyrazolone moiety. Axial chirality associated with central chirality in the product structures was influenced by chiral phosphoric acid catalyst **P23**. To freeze the C–C bond rotation, the pyrazole moiety in **99** required sterically demanding substitutents. Excellent dia- and enantioselective synthesis of the products were caused by a chiral environment induced in the transition state through a dual H-bonding interaction between both the substrates and catalyst. In addition, π–π stacking between the aromatic moieties in both reagents brought more rigidity in the corresponding transition state (Scheme 23) [53].

**Scheme 24:**
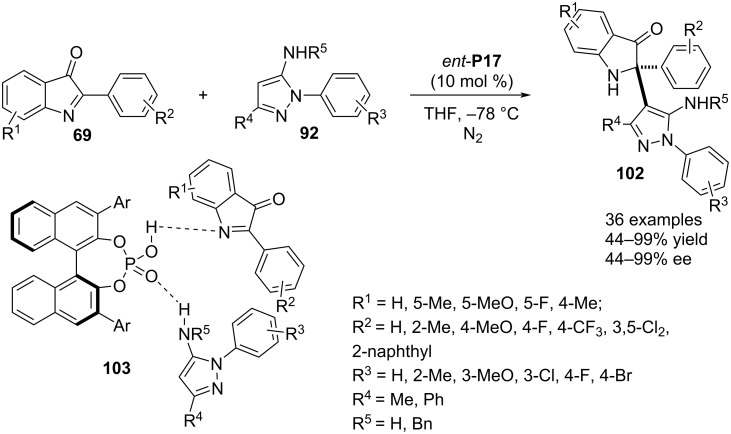
Coupling of 5-aminopyrazole and 3*H*-indol-3-ones.

In 2023, a chiral phosphoric acid *ent*-**P17**-mediated aza-Friedel–Crafts alkylation was reported between 5-aminopyrazole **92** as the π-nucleophile and 3*H*-indol-3-ones **69** as electrophilic reagents. The presence of an amino group in pyrazole **92** is necessary as it is engaged in the H-bonding interaction with the catalyst P=O moiety whereas the imine nitrogen of **69** accepts an H-bond from the catalyst OH group (see transition state **103**). These dual noncovalent interactions were the reason behind a highly face-selective attack by the *ortho*-carbon of the aromatic amine functionality to the cyclic imine allowing a facile access of indolin-3-ones **102** attached to a 5-aminopyrazolyl-substituted aza-quaternary stereocenter via the C2 position. The reaction was very well compatible with various aryl substituents as well as different groups on the benzene ring of indolones **69**. Further broadening of the substrate scope was achieved by changing the aryl substituent attached to the pyrazole ring nitrogen. For enantioenrichment of the products, the presence of a methyl group at the C3 position of the pyrazole ring was obligatory. One example was included with a phenyl substituent at the aforesaid position for which a much diminished enantioselectivity (44%) was obtained (Scheme 24) [54].

### Pyrophosphoric acids

**Scheme 25:**
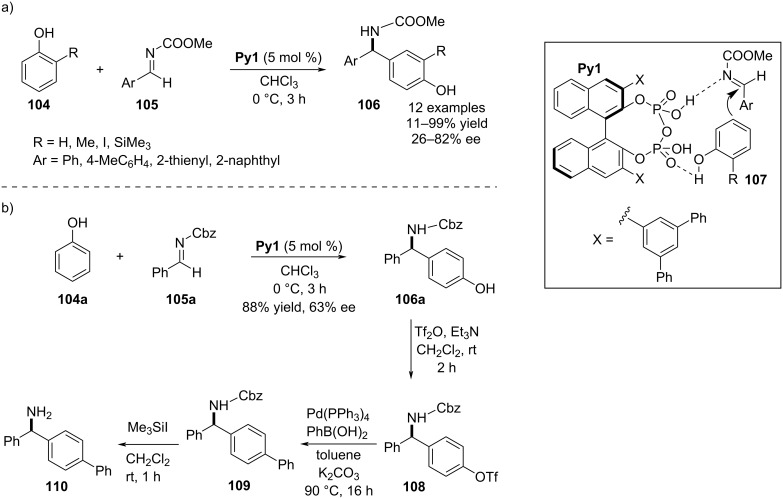
Pyrophosphoric acid-catalyzed aza-Friedel–Crafts reaction on phenols.

In 2018, Ishihara and co-workers demonstrated a highly *para*-selective aza-Friedel–Crafts process using phenols and *ortho*-monosubstituted phenol analogues **104**. As potential electrophiles, *N*-methoxycarbonyl-substituted aldimines **105** were explored to activate the *para*-carbon of the phenol derivatives catalyzed by the chiral pyrophosphoric acid **Py1**. The high regioselectivity was mainly caused by catalyst–substrate interactions via intermolecular H-bonding which could force the π-nucleophile to approach from the less sterically congested *para*-position. As *ortho*-substituents in the phenol derivatives, mainly sterically bulky alkyl, silyl, and iodo groups were incorporated to ensure the complete regioselectivity. On the other hand, various aromatic aldehyde-based aldimines were examined as electrophilic partners. Enantioincorporation into the products was explained by a *Si*-face attack of the nucleophile to the C=N plane. However, this process was not very promising in terms of enantioselectivities (Scheme 25a). The synthetic applicability of this asymmetric process was shown by synthesizing **110**, a key intermediate of (*R*)-bifonazole (Scheme 25b) [55].

### Thioureas and squaramides

**Scheme 26:**
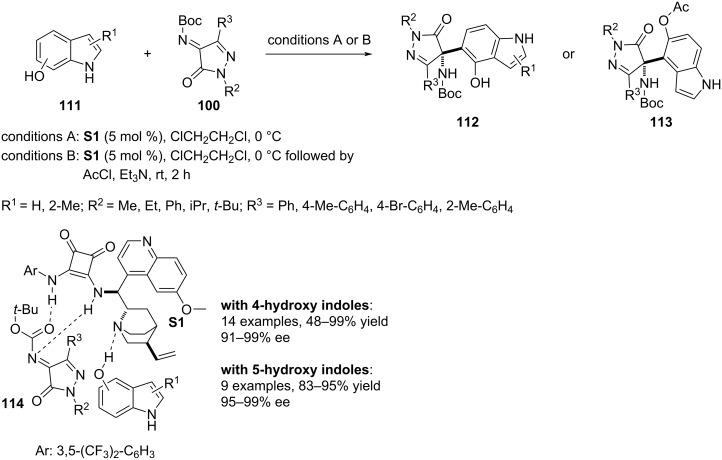
Squaramide-assisted aza-Friedel–Crafts reaction.

In 2018, Yang, Deng and co-workers developed an aza-Friedel–Crafts aminoalkylation of 4- and 5-hydroxyindoles **111**. As electron-demanding component, *N*-Boc pyrazolinone ketimines **100** were investigated to install the all-substituted aza-quaternary stereocenter at the C4 position of the pyrazolinone scaffold. Stereoinduction on this chiral center was regulated by the chiral squaramide catalyst **S1** affording the products with excellent enantioselectivities. A stereodefined transition state organized by triple H-bonding interactions between the catalyst and the substrates controls the enantioefficiency of this process (see transition state **114**). The substrate scope was broader with 4-hydroxyindoles to functionalize the C5–H bond whereas a bit narrower substrate scope was achieved with 5-hydroxyindoles allowing the 4-indolyl-substituted stereocenter formation. In both cases, few more products were added by altering N1 and C3 substituents of **100** (Scheme 26) [56].

**Scheme 27:**
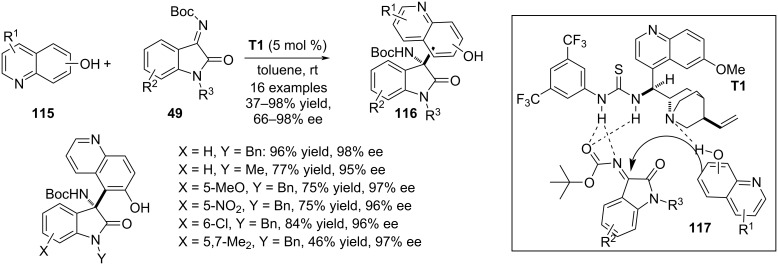
Thiourea-catalyzed aza-Friedel–Crafts reaction.

In the same year, a quinine-derived chiral thiourea-mediated aza-Friedel–Crafts reaction between hydroxyquinolines **115** and isatin-derived ketimines **49** was reported by Vila, Pedro and co-workers. Regioisomeric hydroxyquinolines were tested in this reaction to facilitate the electrophilic aromatic substitution on the *ortho*-carbon atom with respect to the hydroxy group in quinolines **15**. The reaction affords oxindole scaffolds **116** with a hydroxyquinoline-substituted aza-quaternary stereocenter in the 3 position. Most of the examples in this report involved 6-hydroxyquinoline as nucleophile whereas two examples each were presented with 5- and 7-hydroxyquinolines, respectively. Both the imine nitrogen and the carbonyl oxygen of the N-substituted Boc group of **49** were H-bonded with NH groups of the thiourea framework whereas the hydroxy functionality of **116** engaged itself in H-bonding with the quaternary nitrogen of the catalyst (see transition state **117**). These noncovalent interactions were responsible for the stereochemical output of the reaction furnishing the products with moderate to excellent enantioselectivities. Electronically and sterically divergent functionalities in the benzene ring of **49** expanded the substrate scope whereas variation of **115** was very much limited (Scheme 27) [57].

**Scheme 28:**
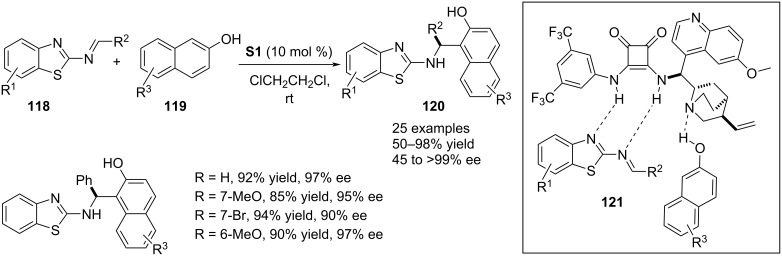
Squaramide-catalyzed reaction between β-naphthols and benzothiazolimines.

In 2021, Wang and co-workers developed an aza-Friedel–Crafts reaction involving β-naphthols **119** as π-nucleophiles and benzothiazolimines **118** as electrophiles. Chiral squaramide **S1**-assisted this process affording enantioenriched 1-((benzothiazol-2-ylamino)methyl)naphthalen-2-ols **120** with high chemical yields. The activation of the electrophile was achieved through acceptance of H-bonds by the nitrogens in **118** from the NH moieties of the catalyst where a free OH group of **119** donated a H-bond to the tertiary amine moiety of **S1**. These noncovalent interactions were responsible for the stereochemical output of the reaction. Different aryl substituents on the imine carbon and functionalities in the carbocyclic ring of **118** were tested. One example was shown with an alkyl-substituted imine which provided the product with much decreased enantioselectivity (45% ee) and four examples were presented by varying the functionalities in the nucleophile (Scheme 28) [58].

**Scheme 29:**
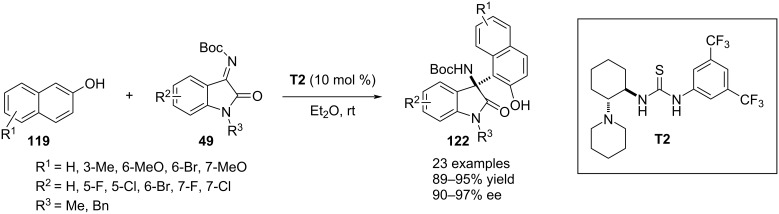
Thiourea-catalyzed reaction between β-naphthol and isatin-derived ketamine.

In 2021, Wang, Jin and co-workers deployed chiral thiourea **T2** as the catalytic agent for executing a highly enantioselective aza-Friedel–Crafts process between β-naphthols **119** and isatin-derived ketimines **49** in the course of accessing enantioenriched 3-amino-2-oxindoles **122** (Scheme 29) [59].

### Other catalysts

**Scheme 30:**
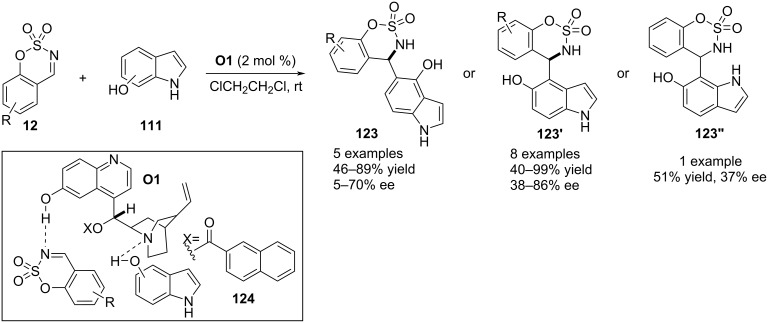
Quinine-derived molecule as catalyst.

In 2019, Vila, Pedro and co-workers reported a functional group-directed activation of the carbocyclic ring of indoles utilizing cyclic imines as electrophiles. The quinine-derived compound **O1** was the catalytic reagent to functionalize the *ortho*-C–H bond of 4-, 5-, and 6-hydroxyindoles **111** via an aza-Friedel–Crafts aminoalkylation involving benzoxathiazine 2,2-dioxides **12** as electron-demanding reagents. H-Bonding engagement of both substrates with the catalyst selectively masked the *Re* face of the imine plane thus forcing the nucleophile to approach from the *Si* face (see transition state **124**, Scheme 30) [60].

**Scheme 31:**
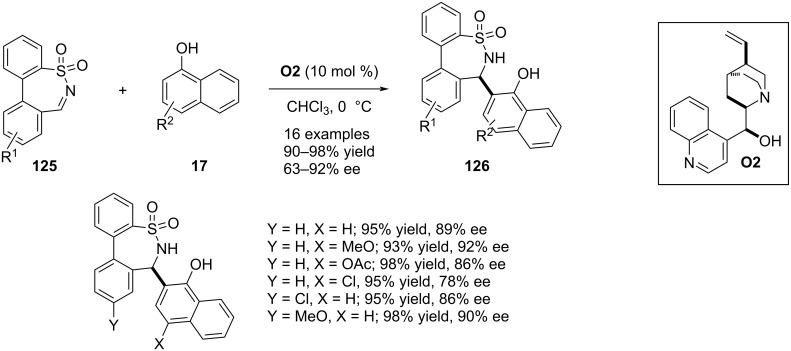
Cinchona alkaloid as catalyst.

In 2019, Zhou and co-workers reported an aza-Friedel–Crafts reaction between α-naphthol derivatives **17** utilizing 7-membered cyclic *N*-sulfonylimines **125** as electrophiles leading to the facile access of ε-sultams **126** bearing a sulfonylamine-substituted stereocenter. Cinchona alkaloid **O2** was the efficient catalyst for this asymmetric C–C bond formation delivering the products with moderate to good enantioselectivities. One example was documented involving β-naphthol as nucleophile and another example included electron-rich phenol (Scheme 31) [61].

**Scheme 32:**
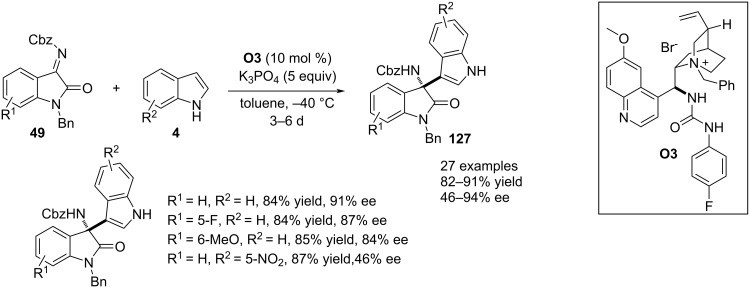
aza-Friedel–Crafts reaction by phase transfer catalyst.

Lin, Duan and co-workers demonstrated an enantioselective aza-Friedel–Crafts reaction between indoles **4** and isatin-derived ketimines **49**. A chiral phase transfer catalyst **O3** derived from urea assisted this organic transformation featuring a C3–H bond functionalization of indoles. Different protecting groups for the imine nitrogen and ring nitrogen of **49** were screened under optimal reaction conditions where Cbz and benzyl were the best protecting groups in terms of enantioselectivities. A product library was prepared by varying sterically and electronic divergent functionalities in the carbocyclic rings of both reactants. Enantioincorporation into the products was explained by H-bonding engagement between the catalyst NHs groups and an ionic interaction between the anionic indole and quaternary ammonium moiety of the catalyst (Scheme 32) [62].

**Scheme 33:**
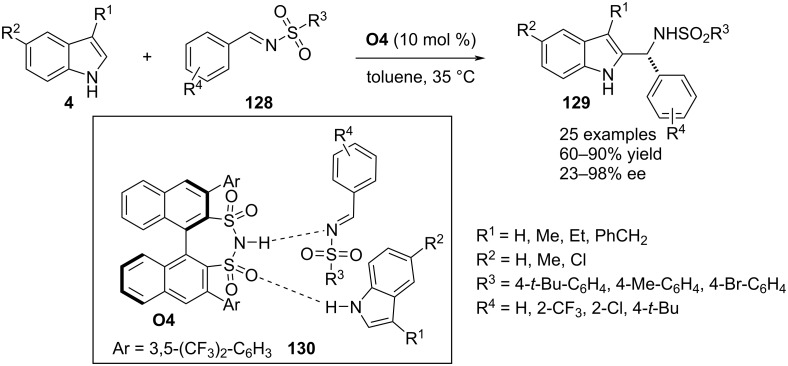
Disulfonamide-catalyzed reaction.

In 2022, Li, Chen and co-workers employed the BINOL-derived chiral disulfonimide **O4** as Brønsted acid catalyst to execute a straightforward aza-Friedel–Crafts reaction between 3-substituted indoles **4** and *N*-sulfonyl-substituted aldimines **128**. The reaction successfully installed an aza-tertiary stereocenter at the C2 position of the heterocyclic ring. A broad substrate scope was investigated by varying substituents on both substrates. A transition state involving dual noncovalent interactions between the catalyst and substrates directed the face-selective addition of the π-nucleophile to the electrophilic carbon of the imine (see transition state **130**, Scheme 33) [63].

### Heterogenous catalysts

**Scheme 34:**
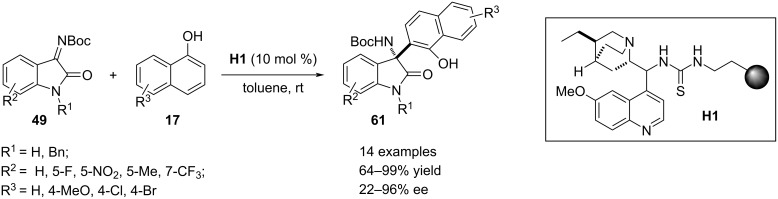
Heterogenous thiourea-catalyzed aza-Friedel–Crafts reaction.

In 2020, Pedrosa and co-workers devised a chiral heterogenous thiourea catalyst that was applied in an enantioefficient aza-Friedel–Crafts process. A series of heterogenous catalysts were prepared by condensation between alkaloids and polystyrene-derived isothiocyanates. These polymer-supported materials were utilized as heterogenous catalyst to execute the aza-Friedel–Crafts reaction between 1-naphthols **17** and isatin-derived ketimines **49** to produce oxindole motif **61** bearing a 1-hydroxynaphth-2-yl-substituted aza-quaternary stereocenter at the C3 position. The best result was obtained with the hydroquinine-based supported catalyst **H1** which efficiently promoted five catalytic cycles without loss of its activity. *N*-Alkyl-substituted ketimines **49** with different functionalities in the benzene ring were well responsive towards the heterogenous reaction to afford the products **61** with moderate to excellent enantiomeric excesses. However, N-unsubstituted **49** (R^1^ = H) resulted in a much diminished stereoselectivity. As the electrophilic partner, isatin-derived ketimine was also utilized which furnished the product with 68% enantiomeric excess. Replacement of the nucleophile in this methodology for substrate scope expansion was carried out by employing 2-naphthol and 4-hydroxyindole (Scheme 34) [64].

### Application in total synthesis

**Scheme 35:**
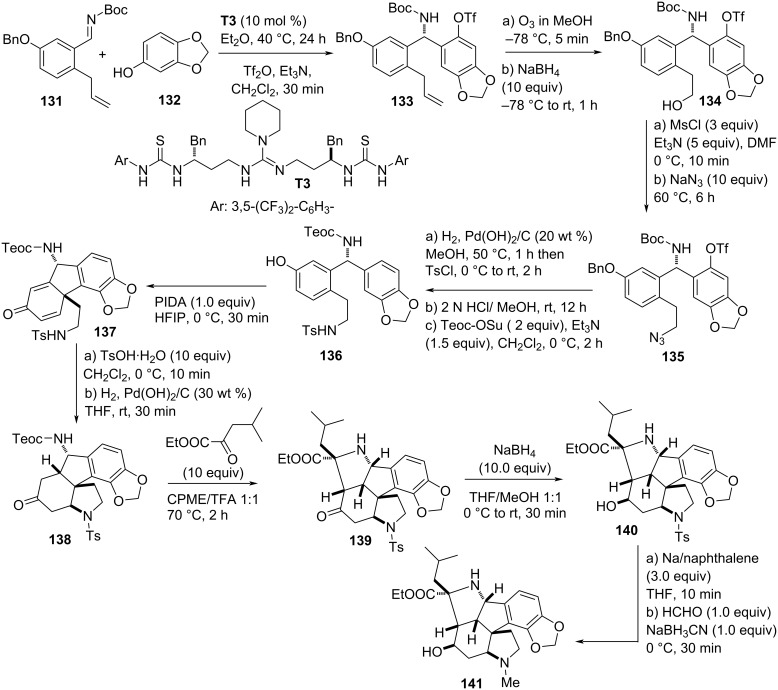
Total synthesis of (+)-gracilamine.

In 2018, a guanidine bisthiourea-catalyzed highly enantioselective aza-Friedel–Crafts reaction was applied as a central step in the total synthesis of (+)-gracilamine. The reaction was designed between sesamol (**132**) and *N*-Boc-protected ketimine **131** in the presence of **T3** as catalyst to introduce the electrophile at the *ortho*-position with respect to the phenolic OH group. The aza-Friedel–Crafts product was obtained with 94% yield and converted into triflate **133** with 74% yield and 99% ee after recrystallization. Subsequent ozonolysis of the terminal alkene functionality with a follow-up reduction furnished primary alcohol **134** which was transformed into the azide **135**. Reduction of the azide **135** was accompanied by debenzylation, was followed by tosylation of the primary amine and exchange of the Boc-protecting group with the Teoc group then gave phenol **136**. Compound **136** was then subjected to a highly diastereoselective oxidative phenolic coupling giving fused tetracyclic architecture **137**. Follow-up acid-mediated intramolecular aza-Michael addition and subsequent alkene reduction provided ketone **138** which was reacted with an α-keto ester in an intramolecular 5-*endo*-*trig*-cyclization process to afford **139**. Treatment of compound **139** with sodium borohydride afforded secondary alcohol **140** which after conversion of the tosyl group into a methyl group gave the final product **141** (Scheme 35) [65].

**Scheme 36:**
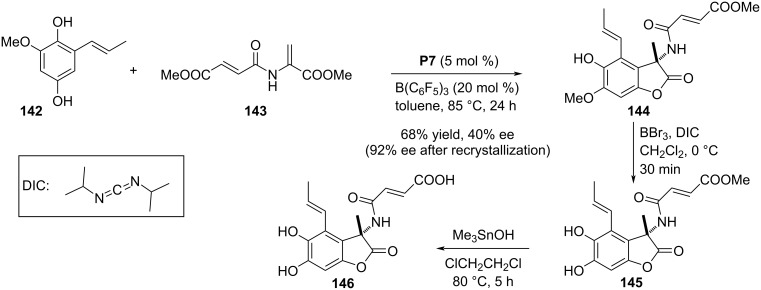
Total synthesis of (−)-fumimycin.

In 2019, Piersanti and co-workers reported an organocatalyzed enantioselective aza-Friedel–Crafts/lactonization domino reaction sequence as the key step in the course of synthesizing (+)- and (−)-fumimycin. (−)-Fumimycin was first isolated from *Aspergillus fumisynnematus* and exhibits antibacterial acitivity against resistant *S. aureus* strains. It is also an inhibitor of the enzyme peptide deformylases (PDFs). The synthesis comprised the reaction between the highly substituted hydroquinone **142** and dehydroalanine **143** in the presence of chiral phosphoric acid **P7** as catalyst to prepare benzofuran-2(3*H*)-one derivative **144** having an aza-quaternary stereocenter. The achiral Lewis acid tris(pentafluorophenyl)borane was required as additive in the reaction system to enhance the chemical yield and enantioselectivity. After two additional steps, i.e., demethylation of the phenolic ether and ester hydrolysis, (−)-fumimycin (**146**) was obtained (Scheme 36) [66].

## Conclusion

The aza-Friedel–Crafts reaction is a powerful reaction that allows the incorporation of an aminoalkyl functionality in aromatic systems through C–C bond formation. This C–H bond functionalization methodology of aromatic systems also has the possibility of incorporating aza stereocenters into a product depending upon the choice of a suitable electrophile, i.e., imines. The present review assembled recent (from 2018 till date) examples of asymmetric versions of this important method mediated by different organocatalysts. The mechanistic approaches with explanation about the origin of stereoselectivities has also been elaborated. This reaction has been successfully utilized as the key step in the syntheses of different important natural products which have been included in this article as well. On searching the literature, it has been found that mainly H-bonding chiral organic molecules have been envisaged as the catalytic systems for stereoinduction into products. The asymmetric induction is caused by effective noncovalent interactions between the catalysts and substrates to force a face-selective attack by the nucleophile, i.e., the aromatic π-system to the electrophile.
